# *Epsc* Involved in the Encoding of Exopolysaccharides Produced by *Bacillus amyloliquefaciens* FZB42 Act to Boost the Drought Tolerance of *Arabidopsis thaliana*

**DOI:** 10.3390/ijms19123795

**Published:** 2018-11-29

**Authors:** Xiang Lu, Shao-Fang Liu, Liang Yue, Xia Zhao, Yu-Bao Zhang, Zhong-Kui Xie, Ruo-Yu Wang

**Affiliations:** 1 Gaolan Station of Agricultural and Ecological Experiment, Northwest Institute of Eco-Environment and Resources, Chinese Academy of Sciences, Lanzhou 730000, China; chenchenai9021@gmail.com (X.L.); 15652794633@163.com (S.-F.L.); 15002517498@163.com (L.Y.); zhaoxia@lzb.ac.cn (X.Z.); lux12@lzu.edu.cn (Y.-B.Z.); wxhcas@lzb.ac.cn (Z.-K.X.); 2 Key Laboratory of Stress Physiology and Ecology in Cold and Arid Regions of Gansu Province, Lanzhou 730000, China; 3 University of Chinese Academy of Sciences, Beijing 100049, China

**Keywords:** *Bacillus amyloliquefaciens* FZB42, biofilm, drought stress, *epsC*, exopolysaccharides, phytohormone

## Abstract

*Bacillus amyloliquefaciens* FZB42 is a plant growth-promoting rhizobacteria that stimulates plant growth, and enhances resistance to pathogens and tolerance of salt stress. Instead, the mechanistic basis of drought tolerance in *Arabidopsis* *thaliana* induced by FZB42 remains unexplored. Here, we constructed an exopolysaccharide-deficient mutant *epsC* and determined the role of *epsC* in FZB42-induced drought tolerance in *A. thaliana*. Results showed that FZB42 significantly enhanced growth and drought tolerance of *Arabidopsis* by increasing the survival rate, fresh and dry shoot weights, primary root length, root dry weight, lateral root number, and total lateral root length. Coordinated changes were also observed in cellular defense responses, including elevated concentrations of proline and activities of superoxide dismutase and peroxidase, decreased concentrations of malondialdehyde, and accumulation of hydrogen peroxide in plants treated with FZB42. The relative expression levels of drought defense-related marker genes, such as *RD29A*, *RD17*, *ERD1*, and *LEA14*, were also increased in the leaves of FZB42-treated plants. In addition, FZB42 induced the drought tolerance in *Arabidopsis* by the action of both ethylene and jasmonate, but not abscisic acid. However, plants inoculated with mutant strain *epsC* were less able to resist drought stress with respect to each of these parameters, indicating that *epsC* are required for the full benefit of FZB42 inoculation to be gained. Moreover, the mutant strain was less capable of supporting the formation of a biofilm and of colonizing the *A. thaliana* root. Therefore, *epsC* is an important factor that allows FZB42 to colonize the roots and induce systemic drought tolerance in *Arabidopsis*.

## 1. Introduction

Tissue dehydration imposed by drought can cause irreversible cellular damage, with a consequential loss in the economic yield of crop plants [1]. A number of breeding approaches are being actively pursued to promote the drought tolerance of many leading crop species. An alternative approach to drought mitigation relies on the presence of certain plant growth-promoting rhizobacteria (PGPR) [2,3,4,5]. Improving the tolerance of drought stress in plants using PGPR and understanding the molecular mechanisms involved can help to address strategic needs and complement classical genetic selection strategies.

Studies have shown that PGPR can promote the growth and development of host plants as well as eliciting induced systemic tolerance to reduce the susceptibility of plants to drought conditions [6,7,8,9,10]. It has been established that their presence affects the host’s metabolism, in particular by boosting its production of osmoprotectants, promoting photosynthesis, and increasing the activity of reactive oxygen species (ROS) scavenging enzymes [11,12,13]. Other studies have determined that their presence regulates a number of phytohormones, such as abscisic acid (ABA), salicylic acid (SA), jasmonic acid (JA), and ethylene (ET), thereby influencing many plant signaling networks, including those involved in the abiotic stress response [14,15,16,17]. 

Bacterial cells proliferate and associate into multicellular structure, form robust floating pellicle in standing medium referred as biofilm at the air–liquid interface [18,19]. Biofilms can protect member cells from adverse environmental stresses [20]. PGPR form biofilms after being inoculated in the rhizosphere of plants [21,22]. The formation of PGPR biofilm is a complex process, which is regulated by multiple genes. It has been revealed that *degU*, *abrB*, and *resE* regulate the formation of *B. amyloliqueliciens* SQR9 biofilm [23,24,25]. *B. amyloliquefaciens* FZB42 collagen-like proteins encoding genes, including *clpA*, *clpB*, *clpC*, and *clpD*, have been reported to be crucial for biofilm formation and adhesion to plant roots [26].

Many microbes, especially those belonging to certain bacterial and fungal species, secrete exopolysaccharides, large carbohydrate polymers formed by the concatenation of monosaccharides through glycosidic bonds [27]. These compounds have been shown to be protective of plants challenged by abiotic stress [28,29]. For instance, in soybean plants exposed to salinity stress, the exopolysaccharides generated by certain *Pseudomonas* spp. act to reduce the translocation of Na^+^ ions from the soil into the root [30]. Consortia of PGPR and their respective exopolysaccharides have been suggested as conferring a greater benefit with respect to drought tolerance compared with inoculation using PGPR alone [31]. Moreover, exopolysaccharides are a large component of bacterial biofilms [32]. Thus, in terms of the beneficial effects of PGPR on their interactions with plants, it is hardly surprising that exopolysaccharides are of great significance in improving the ability of plants to resist drought stress.

Strain FZB42 of the gram-positive bacterium, *Bacillus amyloliqueliciens*, has been shown to promote plant growth by increasing the supply of mineral nutrition to the plant, while also boosting the plants’ level of resistance to some pathogens and their tolerance of salinity stress [33,34,35]. However, to the best of our knowledge, no previous studies have evaluated the effects of FZB42 on drought stress in *Arabidopsis* and study the underlying molecular mechanisms involved. 

Here, a comparison between the impact of inoculation with wild type FZB42 and an *epsC* mutant strain that is incapable of producing exopolysaccharides on the drought tolerance of model plant *Arabidopsis* was observed [36]. We found that FZB42 could induce drought tolerance in *Arabidopsis* via ethylene and jasmonate-mediated pathways and the *epsC* mutant had a decreased capacity for inducing drought tolerance. Thus, we suggest that exopolysaccharides encoding gene *epsC* plays a crucial role in allowing FZB42 to improve the tolerance of drought by *Arabidopsis*.

## 2. Results

### 2.1. Epsc Contributes to the Ameliorative Effect of B. amyloliquefaciens on the Drought Tolerance of A. thaliana

**Figure 1:**
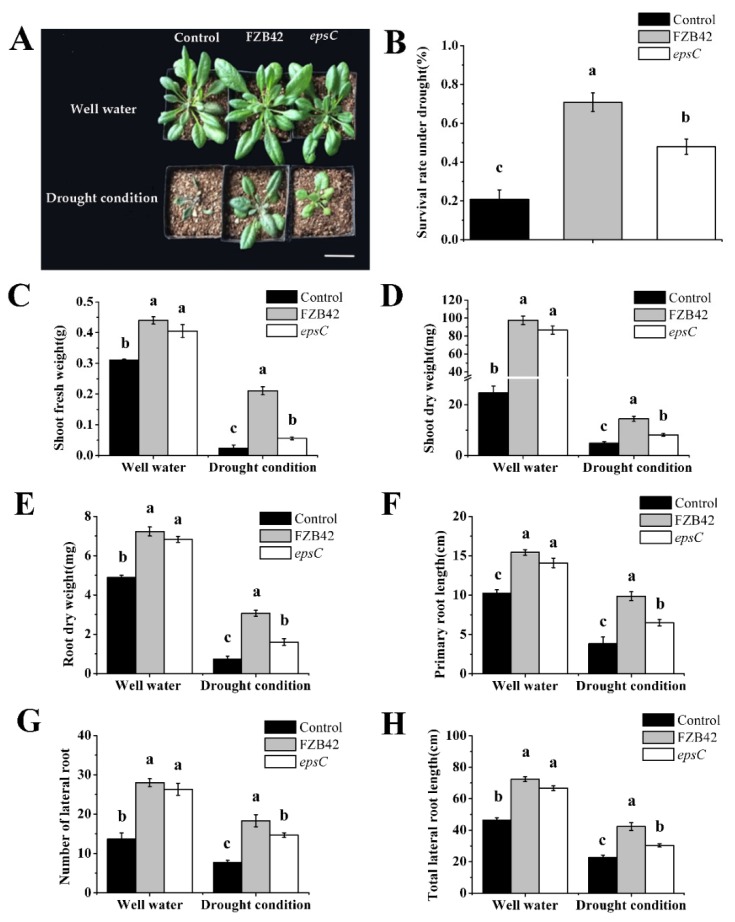
The effect of inoculation with either wild type FZB42 or the *epsC* mutant on the performance of *A. thaliana* seedlings. Appearance of the plants. Scale bar, 2 cm (**A**), seedling survival rate under drought stress (**B**), shoot fresh weight (**C**), shoot dry weight (**D**), root dry weight (**E**), primary root length (**F**), lateral root number (**G**), total lateral root length (**H**). Values in (**B**) through (**H**) are shown as means, with the whiskers representing the standard error (SE, *n* = 18). Different letters above each column indicate statistically significant (*p* < 0.05) differences in mean performance.

Under well-watered conditions, inoculation with either wild type FZB42 or the *epsC* mutant, the shoot biomass of *A. thaliana* seedlings increased significantly (Figure 1A,C,D). Furthermore, the benefit to the plants’ performance was similar for both strains of *B. amyloliquefaciens* (Figure 1C,D). Similarly, we found that treatment with FZB42 and the *epsC* mutant significantly increased root dry weight, primary root length, lateral root number, and total lateral root length in *Arabidopsis* compared with the non-inoculated plants (*p* < 0.05), and there were no significant differences between the FZB42 and *epsC* mutant treatments (Figure 1E–H).

After drought treatment, inoculation with FZB42 and the *epsC* mutant in the rhizosphere of *Arabidopsis* significantly improved survival rate, increased shoot dry weight and fresh weight, affected root system, including increased root dry weight, primary root length, lateral root number, and total lateral root length in *Arabidopsis* seedlings compared with the control treatment (*p* < 0.05) (Figure 1B–H). However, the benefit was less marked when the *epsC* mutant was used for the inoculation. 

### 2.2. The Presence of EpsC Promoted Antioxidant Metabolism in Droughted A. thaliana Plants

**Figure 2:**
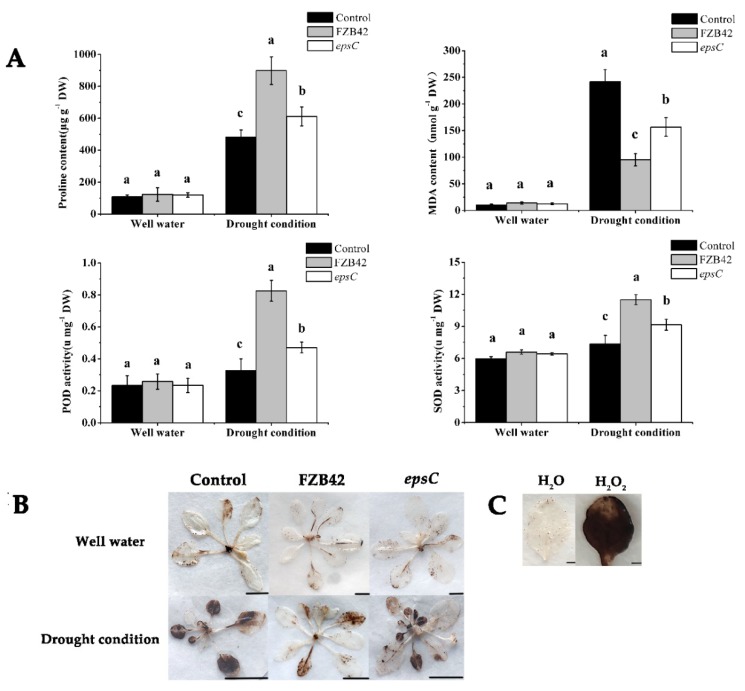
The biochemical response of the *A. thaliana* leaf to drought stress. The concentration of proline and malondialdehyde (MDA), and the activity of superoxide dismutase (SOD) and peroxidase (POD) (**A**), 3,3-diaminobenzidine staining revealing the accumulation of hydrogen peroxide in well-watered and droughted plants inoculated with either wild type FZB42 or the *epsC* mutant. Scale bar, 1 cm (**B**), 3,3-diaminobenzidine staining revealing the accumulation of hydrogen peroxide in wild type Arabidopsis leaves treated with H_2_O or 20 mM H_2_O_2_. Scale bars, 1 mm (**C**). Values in (**A**) are shown as means, with the whiskers representing the standard error (SE, *n* = 18). Different letters above each column indicate statistically significant (*p* < 0.05) differences in mean performance.

Under well-watered conditions, inoculation with either wild type FZB42 or the *epsC* mutant had no effect on the concentrations in the leaf of either proline or malondialdehyde (MDA), nor did it influence the activity of either peroxidase (POD) or superoxide dismutase (SOD) (Figure 2A). Under drought stress conditions, the application of FZB42 and the *epsC* mutant greatly increased the proline contents and the activities of SOD and POD compared with the control (*p* < 0.05) (Figure 2A). However, inoculation with the *epsC* mutant significantly decreased the proline content and the activities of SOD and POD compared with the FZB42 treatment (*p* < 0.05) (Figure 2A). In addition, the MDA content decreased significantly after inoculation with FZB42 and the *epsC* mutant compared with the control treatment (*p* < 0.05) (Figure 2A). However, a greater increase in leaf MDA content was observed in plants that had been inoculated with the *epsC* mutant than those that had been inoculated with the wild type strain (Figure 2A) (*p* < 0.05) (Figure 2A).

We then detected the accumulation of hydrogen peroxide in the leaves under well-watered conditions by DAB (3,3-diaminobenzidine) staining. The accumulation of hydrogen peroxide in the leaves of non-treated plants was similar as in the leaves of plants treated with either the wild type or the mutant strain of *B. amyloliquefaciens* (Figure 2B,C). However, the accumulation of hydrogen peroxide in the leaves of non-inoculated plants under drought stress was more substantial than in the leaves of plants inoculated with either the wild type or the mutant strain of *B. amyloliquefaciens*; and the level was higher in the *epsC* mutant-inoculated ones than in the wild type-inoculated ones. (Figure 2B,C).

### 2.3. The Presence of EpsC Influenced Stomatal Control from Detached Leaves in Droughted A. thaliana Plants

**Figure 3:**
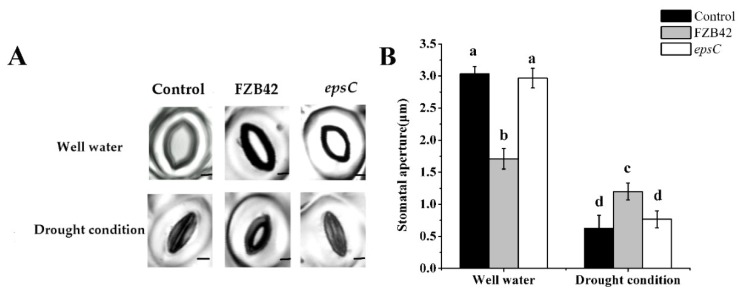
The physiological response of the *A. thaliana* leaf to drought stress. Appearance of stoma. Scale bar, 2.5 µm (**A**), and stomatal aperture in detached leaves of plants inoculated with either wild type FZB42 or the *epsC* mutant (**B**). Values are shown as means, with the whiskers representing the standard error (SE, *n* = 18). Different letters above each column indicate statistically significant (*p* < 0.05) differences in mean performance.

A comparison between the leaves of well-watered plants derived from the three inoculation treatments (non-inoculated and inoculated with either the wild type or the mutant strain of *B. amyloliquefaciens*) suggested that stomatal aperture was smaller in the leaf of plants inoculated with the wild type strain than in those of plants exposed to either of the other two treatments: Stomatal aperture in the leaf of non-inoculated plants was indistinguishable from that seen in the leaf of the mutant strain-inoculated ones (Figure 3). The effect of moisture stress was to greatly reduce stomatal aperture, irrespective of the inoculation treatment. Somewhat unexpectedly, stomatal aperture in the leaf of plants inoculated with the wild type strain was significantly greater than in the leaf of plants exposed to either of the two other treatments; just as in the well-watered plants, stomatal aperture did not respond to inoculation with the mutant strain (Figure 3). 

### 2.4. The Presence of EpsC Altered the Transcript Levels of Stress-Responsive Genes in Arabidopsis Plants 

**Figure 4:**
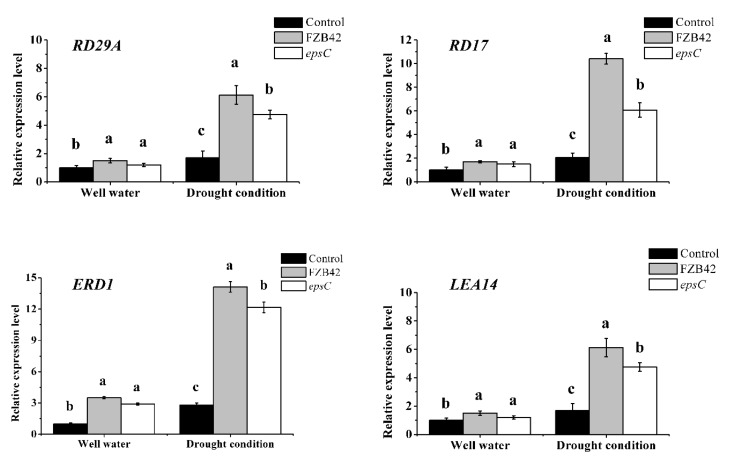
The relative expression levels of drought-responsive marker genes in the leaves of *A. thaliana*. A qRT-PCR assay was used to estimate the expression levels of the stress response-associated marker genes, *RD29A*, *RD17*, *ERD1*, and *LEA14*, in drought plants inoculated with either wild type FZB42 or the *epsC* mutant. Values are shown as means, with the whiskers representing the standard error (SE, *n* = 18). Different letters above each column indicate statistically significant (*p* < 0.05) differences in mean performance.

The transcription levels of stress-responsive marker genes, *RD29A*, *RD17*, *ERD1*, and *LEA14*, in the leaves were assessed using qRT-PCR (Figure 4). All of these genes are involved in the typical response to drought. Compared with the expression level of the genes in control *Arabidopsis* plants (without bacterial inoculation), the abundance of each of the transcripts were significantly higher in *Arabidopsis* plants inoculated with FZB42 or the *epsC* mutant (*p* < 0.05) (Figure 4). Of note was that the extent of the induction was uniformly greater in the leaf of plants inoculated with the wild type strain FZB42 than in leaves from plants inoculated with the mutant strain (Figure 4).

### 2.5. The Additional Drought Tolerance Induced by Inoculation with FZB42 Involved the Action of both Ethylene and Jasmonate 

**Figure 5:**
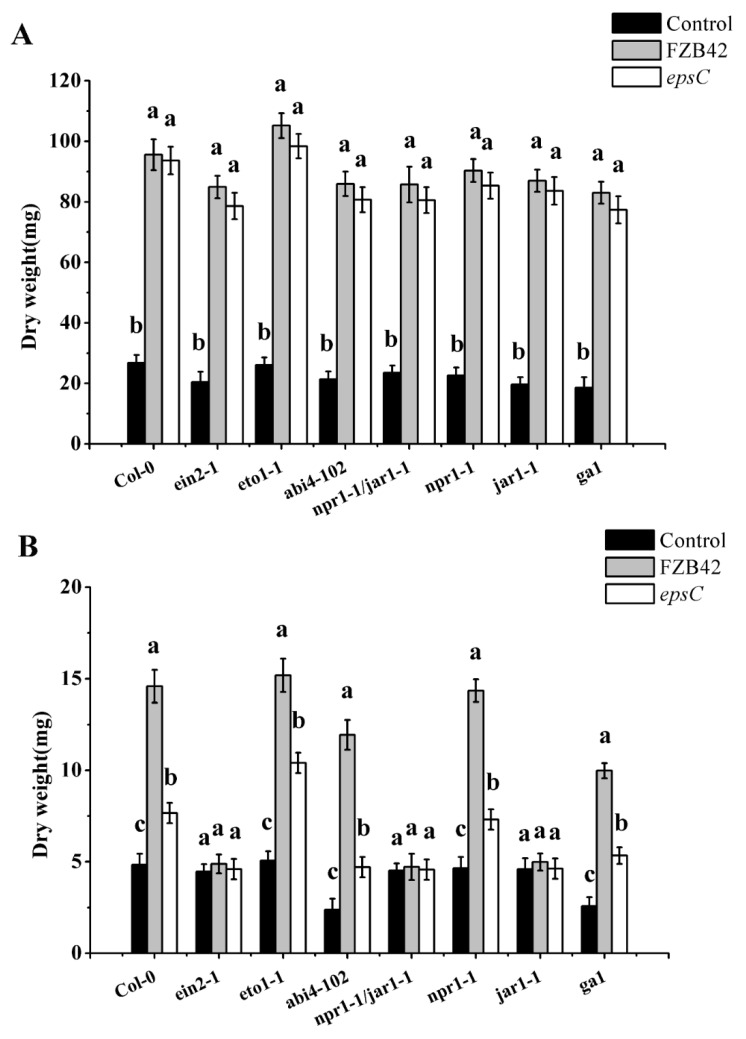
The effect of inoculation with either wild type FZB42 or the *epsC* mutant on the accumulation of dry matter by well-watered and drought seedlings of *Arabidopsis* mutants *ein2-1* (ethylene insensitive), *eto1-1* (ethylene over-producer), *jar1-1* (jasmonate insensitive), *abi4-102* (ABA insensitive), *ga1* (gibberellin-responsive male-sterile dwarf), *npr1-1* (salicylic acid insensitive), and the *npr1-1/jar1-1* double mutant. Dry weight of *Arabidopsis* mutants under well-watered condition (**A**), dry weight of *Arabidopsis* mutants under drought condition (**B**). Values are shown as means, with the whiskers representing the standard error (SE, *n* = 18). Different letters above each column indicate statistically significant (*p* < 0.05) differences in mean performance.

A series of established *A. thaliana* mutants involving altered responses to the major phytohormones were then tested for their interaction with FZB42 and the *epsC* mutant: The mutants were *ein2-1* (ethylene insensitive), *eto1-1* (ethylene over-producer), *jar1-1* (jasmonate insensitive), *abi4-102* (ABA insensitive), *ga1* (gibberellin-responsive male-sterile dwarf), *npr1-1* (salicylic acid insensitive), and the *npr1-1/jar1-1* double mutant. When treated with wild type FZB42 and the *epsC* mutant cells under the well-watered condition, all the mutant plants exhibited a significantly higher dry weight than untreated plants. Furthermore, the dry weight of mutant plants was similar for both strains of *B. amyloliquefaciens* (Figure 5A).

However, when *eto1-1*, *abi4-102*, *npr1-1*, and *ga1* plants were inoculated with wild type FZB42 and the *epsC* mutant cells, the plants exhibited a significantly higher dry weight under drought condition than did the equivalent non-inoculated plants. In contrast, the shoot dry weight of *ein2-1*, *jar1-1*, and the *npr1-1/jar1-1* double mutant did not change when inoculated with either strain (Figure 5B). 

### 2.6. The Absence of epsC Impaired Biofilm Formation and Compromised the Colonization of Arabidopsis Roots 

**Figure 6:**
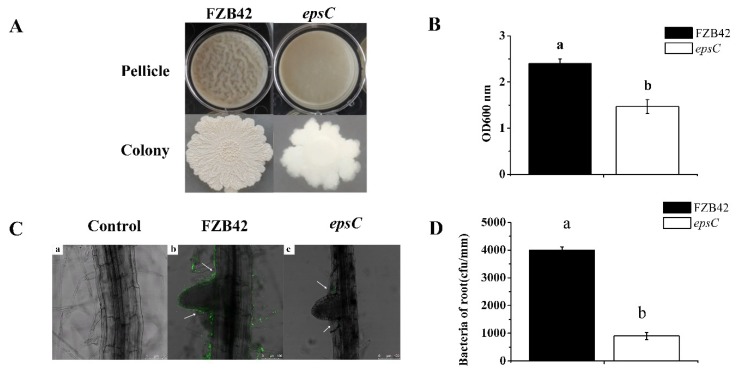
Biofilm formation by wild type FZB42 and the *epsC* mutant. The upper panel illustrates the formation of a pellicle by cells cultured at 30 °C for three days on LBGM medium (Lysogeny broth supplemented with 1% (*v*/*v*) glycerol and 0.1 mM MnSO_4_); the lower panel illustrates colony morphology (**A**), quantitative spectrophotometric assay of biofilms stained with crystal violet (**B**), confocal laser scanning microscopy analysis of root colonization. Scale bar, 100 µm (**C**), adherence capacities of wild type and *epsC* mutant to the *A. thaliana* root (**D**). Values in (**B**) and (**D**) are shown as means, with the whiskers representing the standard error (SE, *n* = 12). Different letters above each column indicate statistically significant (*p* < 0.05) differences in mean performance.

To confirm the roles of *epsC* in biofilm formation by FZB42, we determined the biofilm formation activities of the wild type and *epsC* mutant strain. The wild type and *epsC* mutant strain produced colonies with different shapes and pellicle characteristics on biofilm growth-specific LBGM medium (Lysogeny broth supplemented with 1% (*v*/*v*) glycerol and 0.1 mM MnSO_4_) (Figure 6A). The wild type strain was more effective than the mutant strain in forming a biofilm. Furthermore, the crystal violet staining procedure was used to quantify the amount of biofilm formed confirmed the superiority of the wild type strain (*p* < 0.05) (Figure 6B). 

Inoculation with *GFP*-labeled strains made it possible to monitor the progress of root colonization via confocal laser scanning microscopy (CLSM). This analysis demonstrated that the wild type strain was able to colonize the entire root surface, including the lateral roots, while the mutant strain exhibited a weaker capacity to colonize (Figure 6C). Consistently, in seedlings co-cultivated with *B. amyloliquefaciens* strains for seven days, the adherence capacity of mutant strain *epsC* was significantly lower compared with that of wild type FZB42 (*p* < 0.05) (Figure 6D).

### 2.7. Composition of the Exopolysaccharides Produced by B. amyloliquefaciens FZB42

**Figure 7:**
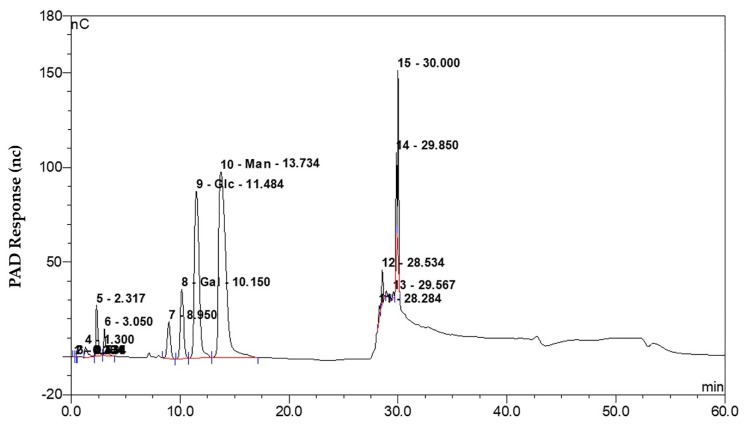
High performance anion exchange chromatography analysis of the monosaccharide composition of exopolysaccharides formed by wild type FZB42 cells. Peaks 8, 9, and 10 correspond to, respectively, galactose, glucose, and mannose. Red line represented the base line. Additional signals occurring between 28 and 32 min were system peaks.

Investigating the monosaccharide compositions of exopolysaccharides is important for establishing their functional relationships. Applying high performance anion exchange chromatography to reveal the composition of the exopolysaccharides produced by FZB42 cells showed that it was composed of the three monosaccharides mannose, galactose, and glucose. The molar ratio of these three compounds was 19.0:2.3:1.0 (Figure 7).

## 3. Discussion

Efforts to improve the tolerance of plants to drought have focused on a range of traits, most notably their capacity to access water from the soil by either enlarging their root system or by enhancing the production of osmolytes, and by the control of stomatal aperture. Here, it has been shown that the drought response of *A. thaliana* was boosted by colonization with *B. amyloliquefaciens* FZB42 (Figure 1). Similar results were obtained after inoculating foxtail millet with *Pseudomonas fluorescens* [37], wheat with *Bacillus subtilis* LDR2 [38], and *Arabidopsis* and maize with *Mitsuaria* sp. and *Burkholderia* sp. [39] under water deprivation conditions. A universal component of the plant response to drought stress is the accumulation of ROS, compounds that are cytotoxic if allowed to build up to an excessive level, and which therefore need to be neutralized [40,41]. Elevated levels of proline and high activities of POD and SOD in plants may be correlated with enhanced stress tolerance [42,43,44]. The presence of certain *Bacillus* sp. is known to support the ability of moisture-stressed plants to increase their cellular content of the osmolyte proline and the activity of ROS scavenging enzymes, such as POD and SOD [45,46], which was precisely the consequence of inoculating *A. thaliana* with wild type FZB42 (Figure 2A). At the same time, the inoculation reduced the cellular production of MDA, a marker of cell membrane damage [14,47], as similarly observed when droughted *A. thaliana* plants were inoculated with *Azospirillum brasilense* [48]. The accumulation of hydrogen peroxide was also lower in the leaf of plants inoculated with FZB42 (Figure 2B). The implication is that FZB42 cells are able to mitigate against lipid peroxidation, thereby helping to retain the integrity of the cell membranes in the leaf of droughted *A. thaliana* seedlings. The stomatal aperture is a major factor that contributes to drought tolerance [49,50,51]. In our investigation, under well-watered conditions, a negative effect of FZB42 inoculation was observed on stomatal conductance compared with the non-inoculated control. However, the application of FZB42 to *Arabidopsis* increased the stomatal aperture under drought conditions (Figure 3). The result is supported by a stronger stomatal closure inhibition after inoculation of soybean, *Platycladus*, and maize with *Bacillus* sp. [52,53,54]. Therefore, inoculation with FZB42 had beneficial effects on seedling growth in *Arabidopsis* even when plants were exposed to severe drought stress condition.

The products of many genes have been implicated as contributing to the survival of *A. thaliana* plants challenged with moisture deficiency [55]. The transcriptional outcome of some of these of inoculating with FZB42 was for *RD29A, RD17, ERD1*, and *LEA14* all to be up-regulated (Figure 4), as has similarly been observed in cognate studies [56,57,58,59]. Considering the physiological changes, this indicated FZB42 could induce systemic drought tolerance in *A. thaliana*. While the direct molecular basis of the FZB42 effect is unknown, it is likely that phytohormone-mediated processes are involved, since these are central to the plant abiotic stress response [60,61,62]. Several studies have shown that the expression of *RD29A, RD17, ERD1*, and *LEA14* are associated with drought tolerance in *A. thaliana* in an ABA independent way [63,64]. Consistently, the drought response (in terms of dry matter accumulation) of a set of key *A. thaliana* mutants suggested that the FZB42 did not act through pathways controlled by either ABA, gibberellin, or salicylic acid, but jasmonate and ethylene (Figure 5B). The non-involvement of ABA was also supported by the stomatal movement data, which showed that stomatal aperture in droughted plants was greater in FZB42-inoculated plants than in non-inoculated ones. However, all mutant plants inoculated with wild type FZB42 cells under the well-watered condition exhibited a significantly higher dry weight than did the equivalent non-inoculated plants (Figure 5A). This suggests the growth-promoting effects and drought tolerance induction of FZB42 on plants may not be the same signaling pathway. Thus, the underlying molecular processes responsible for enhancing cell viability and growth still need to be elucidated.

Microbial exopolysaccharides have been suggested as being able to promote plant growth, along with improving tolerance/resistance to both abiotic and biotic stress [29]. When the *B. subtilis sacB* gene was expressed in *Nicotiana tabacum* and in *Beta vulgaris*, in both cases, there was a measurable improvement in the plants’ tolerance of drought [65,66], thought to be engendered by an alteration in fructan metabolism. The fructans act to protect plants from dehydration by interacting directly with the cell membrane. When certain rhizobacteria are genetically modified to over-produce trehalose, their ability to support the survival of the host plant under conditions of severe water limitation appears to be bolstered [67]. *Rhizobium* sp. cells impaired for the ability to produce extracellular polysaccharide are less able than wild type cells to buffer their plant host against oxidative stress. Here, the effect of removing the capacity to generate exopolysaccharides strongly compromised the ability of *B. amyloliquefaciens* to protect *A. thaliana* from drought stress: Compared to those seedlings inoculated with the wild type FZB42 strain, seedlings treated with the mutant strain showed a lower survival rate, accumulated less biomass, and suffered from more extensive oxidative stress. Nevertheless, the performance of these seedlings was still superior to that of non-inoculated seedlings, which implied that the improved drought tolerance imparted by wild type cells did not just result from the presence of the bacterial exopolysaccharides. It is known in this context that certain volatile organic compounds can also mediate plant abiotic stress tolerance [68,69]. Moreover, the beneficial effect was not lost in the *jar1-1* mutant or the *ein2-1* and *eto1-1* mutant, which suggests that the *epsC* in FZB42 might not participate in the regulation of the jasmonate and ethylene pathway in *Arabidopsis* (Figure 5B).

Exopolysaccharides represent a major component of bacterial biofilms [70,71,72]. Biofilms are usually produced by a multicellular assemblage and are critical to both adhesion and colonization [73,74]. It has been documented that biofilms help microbes tolerate adverse environmental conditions and that they are required to maintain a long-term interaction between the microbe and its plant host [75,76]. Here, it was demonstrated that the product of *B. amyloliquefaciens epsC* mediated biofilm formation and was influential in the process of root colonization (Figure 6). Hence, we suggest that *epsC* is important for the colonization of the rhizosphere of *Arabidopsis* and the induction of drought tolerance by FZB42. Given that *epsC* is responsible for the production of exopolysaccharides, we hypothesize that exopolysaccharides may comprise a microbe-associated molecular pattern that is recognized by plants to induce drought tolerance. The exopolysaccharide produced by FZB42 were shown to be composed of galactose, glucose, and mannose (Figure 7). However, peak 7 could not be identified according to the known monosaccharide, and needs further research. This composition likely determines the structure of the exopolysaccharide, which may ultimately influence its functionality. The focus of this research is expected to shift towards investigating how exopolysaccharides are perceived by the plants and how the plant’s defense response is activated post recognition.

## 4. Materials and Methods

### 4.1. Plant Materials

The experiments were based on the Col-0 ecotype of *A. thaliana*. In addition to wild type, the following mutant lines were also assessed for their response to drought stress: *Ein2-1* (CS3071), *eto1-1* (CS3072), *jar1-1* [77], *abi4-102* (CS3837), *ga1* (CS3103), *npr1-1* (CS3726), and the *npr1-1/jar1-1* double mutant [78] were provided by Nicole K. Clay. The reference stock numbers of *Arabidopsis* mutant lines are deposited in (https://www.arabidopsis.org/abrc/catalog/mutant_seed_28.html. 16 November 2018).

### 4.2. Bacterial Strains and Plasmids 

**Figure 8:**
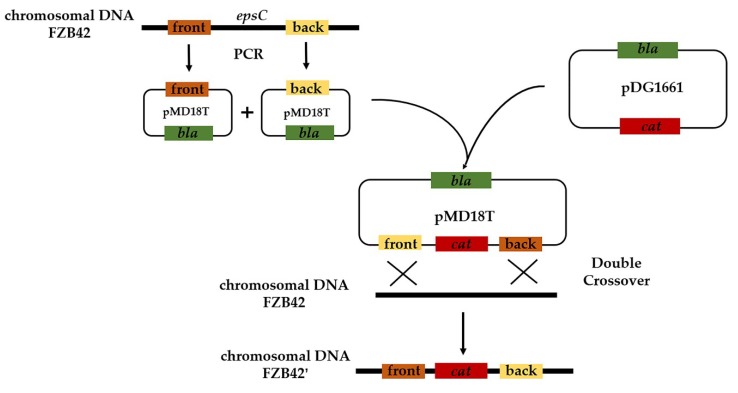
Construction of *epsC* double-crossover deletion mutants.

The bacterial strains used were *B. amyloliquefaciens* FZB42 (BGSC 10A6), a newly created *epsC* mutant (*ΔepsC::Cmr*) and *Escherichia coli* DH5α. All bacteria were cultured at 37 °C in Luria broth (LB) medium. The three plasmids deployed were pDG1661, containing *Chlor(R)* (BGSC ECE112); pMD18-T, containing *Amp(R)* (TaKaRa, Kusatsu, Japan), and pMD18-*epsC*, a derivative of pMD18-T (Figure 8).

### 4.3. Construction of an epsC Mutant in FZB42

The *epsC* gene was disabled via double crossover homologous recombination. Two fragments of the intact gene were PCR-amplified from a template of wild type FZB42 chromosomal DNA using ExTaq DNA polymerase (TaKaRa, Kusatsu, Japan) and the primer pairs, respectively, *epsC* front-F/R and *epsC* back-F/R (Table 1). The amplicons were each inserted into pMD18-T, using its HindIII and BamHI; SphI and XbaI cloning sites, and the resulting engineered plasmids introduced into wild type FZB42; transgenic cells were selected on LB (Lysogeny broth) agar plates containing 50 µg/mL chloramphenicol and verified using a PCR targeting a chloramphenicol resistance gene sequence. Wild type FZB42 cells were rendered competent using an established protocol [79], applying the following modifications: Briefly, bacteria were grown overnight in LB medium at 37 °C (200 rpm) and diluted 1:50 in 20 mL of SPI (Spizizen’s minimal medium). The bacteria were then incubated at 37 °C with vigorous shaking (200 rpm) until the middle of the exponential growth period (OD_600_ = 1.3). The culture was then diluted (1:10) in 6 mL SPII (SPI medium supplemented with 1% (*v*/*v*) 50 mM CaCl_2_ and 250 mM MgCl_2_). The cells were then incubated for another 1.5 h at 100 rpm. Next, 60 μL of 10 mmol/L EGTA (ethylene glycol tetraacetic acid) was added to the medium, before shaking for another 10 min. The culture was divided into equal volumes and no more than 5 μL pMD18-*epsC* plasmid DNA was added. After incubation at 37 °C with shaking at 100 rpm for 0.5 h, 1 mL of LB medium containing a sublethal concentration (0.1 μg/mL) of chloramphenicol was added. The cells were cultured with vigorous shaking for 90 min and plated onto selective agar plates. The pMD18T-*epsC* plasmid was also transferred into a derivative of FZB42 harboring GFP [80].

### 4.4. Analysis of the Monosaccharide Composition FZB42 Exopolysaccharide 

Exopolysaccharides were prepared from wild type FZB42 cells following Liu et al. [81]. Briefly, marine LB medium was incubated with 2% inoculum at 30 °C and 200 rpm for 2 days. Next, the exopolysaccharides in the supernatant from the culture were precipitated with chilled absolute ethanol. The precipitate was dissolved in distilled water. Proteins in the exopolysaccharide solution were removed using the Sevag method [82], and small molecular carbohydrates were removed by dialyzing the exopolysaccharide solution against distilled water. The polysaccharide content of the exopolysaccharide solution was determined using the phenol-sulfuric acid method.

The monosaccharide composition was analyzed by HPAEC (ICS5000, Dionex, Sunnyvale, CA, USA) [83]. Ten milligrams of exopolysaccharide were hydrolyzed with 2 mL of 2 mol/L trifluoroacetic acid at 110 °C for 12 h. The hydrolyzate was co-concentrated repeatedly with methanol to dryness and then filtered through a 0.45 μm nylon filter. The standard sugars were prepared in the same manner. The samples were analyzed on a Carbo Pac PA20 column (ID 3 mm × 150 mm) (Dionex, Sunnyvale, CA, USA) with pulsed amperometric detection using the gradient elution procedure with H_2_O–250 mM NaOH–1.0 M NaAc as the mobile phase. The column was eluted at a flow rate of 0.5 mL/min and the injection volume of the sample was 20 μL.

### 4.5. Biofilm Formation and Growth of FZB42 and the epsC Mutant

Biofilms were generated from both wild type FZB42 and *epsC* mutant cells, following the protocol given by Zhao et al. [26], applying the following modifications: Briefly, cells were cultured at 37 °C and grown to OD_600_ = 1.4, before diluting to 1:100 in LBGM liquid medium containing chloramphenicol in a 12-well plate and then incubating at 30 °C for 48 h. LBGM is composed of LB with supplementation of 1% glycerol (*v*/*v*) and 100 μM MnSO_4_ [84]. To determine the colony morphology, 5 μL of cells were plated onto LBGM solid medium and incubated at 30 °C for 72 h. To quantify biofilm growth, we applied the crystal violet staining method in 96-well polystyrene plates (Thermo Fisher Scientific, Waltham, MA, USA). The culture (1:100) was added to 100 μL of LB liquid medium in each well and incubated at 30 °C for 24 h, before staining with 40 μL of 0.25% crystal violet for 15 min and washing three times with phosphate-buffered saline. Next, 200 μL of 95% ethanol was added to dissolve the biofilm for 15 min at room temperature. The biofilm formation in each well was quantified by measuring the OD_600_ using a spectrophotometer. 

### 4.6. Inoculation of A. thaliana with B. amyloliquefaciens

Seed was germinated on solidified Murashige & Skoog medium (MS medium) for one week, after which the seedlings were transplanted into either clean soil or soil inoculated with ~10^7^ CFU·mL^−1^ of either wild type FZB42 or the *epsC* mutant. After 3 days of transplantation, the plants were subjected to experimental treatments for three weeks. *A. thaliana* plants were raised in a chamber delivering a 16 h photoperiod, with the temperature set to 22 ± 2 °C during the light phase and 20 ± 2 °C during the dark phase.

### 4.7. Adherence to and Colonization of A. thaliana Roots by Wild Type FZB42 and the epsC Mutant Cells 

*A. thaliana* seedlings were raised in 12 well microtiter plates containing half strength MS, and inoculated with ~10^6^ CFU·mL^−1^ *B. amyloliqueliciens*. To quantify colonization of the *A. thaliana* seedlings, the roots were removed from seven day old seedlings, then vortexed for 5 s in an Eppendorf tube containing 1 mL sterile saline; the sample was then transferred to a fresh tube containing 1 mL sterile saline and vortexed for a further 20 s. The solution was then diluted three times with saline (1:10, 1:100, 1:1000) and 100 µL samples were plated on plates. After an overnight incubation at 37 °C, a count was taken of the number of *B. amyloliqueliciens* colonies formed. Root colonization was monitored by immersing *A. thaliana* seedling roots for seven days in ~10^6^ CFU mL^−1^ of either wild type FZB42 or the *epsC* mutant cells, after which the roots were rinsed in sterile saline and imaged by confocal laser scanning microscopy (488 nm emission). 

### 4.8. Stomatal Aperture Measurement

*A. thaliana* seedlings were inoculated with either *epsC* mutant or wild type FZB42, and grown under well-watered or drought stress conditions by withholding water. After three weeks, rosette leaves were sampled. The leaf surface imprint method was used to assess stomatal aperture, as described previously [50]. 

### 4.9. Physiological Assays

SOD activity in the leaf was determined following [85] and that of POD using the guaiacol oxidation method [86]. Leaf proline content was obtained from ethanolic extracts prepared by homogenizing ~100 mg tissue in 1 mL 70% ethanol [87], while that of MDA was determined using a reaction based on 2-thiobarbituric acid, as described by [88]. The accumulation of hydrogen peroxide in *A. thaliana* leaves was detected by staining with 3,3-diaminobenzidine, as described elsewhere [89] with minor modifications: Leaf samples were vacuum-infiltrated with 0.1 mg·mL^−1^ 3,3-diaminobenzidine in 50 mM Tris-acetate buffer (pH 5.0), and incubated at room temperature in the dark for 48 h. The reactivity of 3,3-diaminobenzidine with H_2_O_2_ in the experimental conditions was confirmed by infiltrating rosettes leaves for 48h with the 3,3–diaminobenzidine solution buffer in the presence and absence of 20 mM H_2_O_2_.

### 4.10. Quantitative Real Time PCR (qRT-PCR) Analysis 

Total RNA was isolated from *A. thaliana* seedlings using the Tri-Reagent (Sigma, St. Louis, MO, USA), and treated with DNase to remove DNA contaminants. The first cDNA strand was synthesized from a 1 µg aliquot of the resulting RNA by priming with oligo dT in a 20 µL reaction based on a PrimeScript^TM^ RT reagent Kit, following the manufacturer’s protocol. The resulting cDNA was diluted in four volumes of deionized water for use as a qRT-PCR template. The qRT-PCRs were based on SYBR^®^ Premix Ex Taq ™ II (TaKaRa, Japan), with each sample represented by three replicates. The *ACT2* sequence (GenBank: *AT3G18780*) was chosen as the reference. The relevant primers were chosen according to [64,90]. The cycling regime comprised an initial denaturation of 95 °C/30 s, 40 cycles of 95 °C/ 5 s, 60 °C/30 s, followed by melt curve analysis at 95 °C for 15 S, 60 °C for 1 min, and 95 °C for 15 s. Transcript abundances were calculated using the 2^−ΔΔ*C*t^ method [91] and are expressed in the form of mean ± standard error. A one-way analysis of variance was performed to identify significant differences in transcript abundance between plants (either non-stressed or drought-stressed) inoculated with wild type FZB42 or the *epsC* mutant. Means were compared using Duncan’s multiple range test, applying a significance threshold of 0.05, carried out with routines implemented in SPSS software v16.0. 

## Figures and Tables

**Table 1:** Primers used in this study.

Primer	Sequence (5′–3′)	Size of DNA Sequence (bp)	Gene
*epsC* front-F	CCCAAGCTTCGTTGTCCTGAATGATCCGT	539	*epsC* front
*epsC* front-R	CGGGATCCGGAGAACCCGTCAAAATCGTC	……	……
*epsC* back-F	ACATGCATGCGATTTCCCGCGGAAGAAACG	559	*epsC* back
*epsC* back-R	CTAGTCTAGACCAATACGGGGTGTTCCACA	……	……
Chlor-F	CGGGATCCTAGAAGCTTATCGAATTCTCATG	1250	*chlor*
Chlor-R	ACATGCATGCAAGGAGATGGCGCCCAAC	……	……
